# Influence of Different Industrial Agglomeration Modes on Eco-Efficiency in China

**DOI:** 10.3390/ijerph182413139

**Published:** 2021-12-13

**Authors:** Xiaohu Li, Xigang Zhu, Jianshu Li, Chao Gu

**Affiliations:** 1 School of Geographic and Oceanographic Sciences, Nanjing University, Nanjing 210023, China; 2 School of Architecture and Urban Planning, Nanjing University, Nanjing 210093, China; ljs970481937@gmail.com; 3 School of Business, Nanjing University, Nanjing 210093, China; njugc@smail.nju.edu.cn

**Keywords:** industrial agglomeration modes, specialized agglomeration, related diversified agglomeration, unrelated diversified agglomeration, industrial eco-efficiency, city size

## Abstract

It is a key issue for the Chinese government to improve eco-efficiency and realize green development. As a spatial organization mode of industrial labor division, industrial agglomeration has a complex impact on eco-efficiency. However, it is still debatable which industrial agglomeration modes have a positive impact on eco-efficiency. This paper employs a panel threshold model, enterprise micro-level data, and relevant economic environment data from 283 cities in China from 2004 to 2012. It tests the nonlinear effects of specialized, related diversified, and unrelated diversified agglomeration on industrial eco-efficiency. The results show that the impact of specialized and related diversified agglomeration on industrial eco-efficiency is first inhibited and then promoted. The unrelated diversified agglomeration has a significantly negative impact on industrial eco-efficiency, but the negative impact weakens when agglomeration reaches a certain level. Furthermore, the impact of the three agglomeration modes on industrial eco-efficiency depends on city size. The impact of specialized agglomeration on industrial eco-efficiency is insignificant in small- and some medium-sized cities, but it has a significant inhibitory effect on industrial eco-efficiency when the city surpasses medium size. The role of related diversified agglomeration in promoting industrial eco-efficiency is further enhanced with the growth of city size. The impact of unrelated diversified agglomeration on industrial eco-efficiency gradually changes from negative to positive, but it plays a promoting role only when the city reaches the scale of super-large and mega-cities. Finally, this paper suggests that policymakers should formulate differentiated agglomeration policies according to changes in industrial agglomeration level or city size to improve industrial eco-efficiency.

## 1. Introduction

After the Chinese economic reform in 1978, China’s economy expanded rapidly and became the world’s second-largest economy. Simultaneously, China ranks as one of the countries with the most consumption of resources and energy and the highest emission of various pollutants in the world. As the pillar of the national economy, the industrial sector is the main source of China’s economic growth. However, industrial development causes high consumption and emissions, making it the main source of resource and energy consumption and environmental pollution [1,2]. For this reason, the Chinese government has gradually adopted some policies to promote green development. The Chinese government first put forward the strategy of ecological civilization in 2007. The outline of the 13th Five-Year plan in 2015 proposed the concept of green development, emphasizing the gradual transformation to a green development mode of low energy consumption, low emissions, and high yield. The report of the 19th National Congress also clearly proposed to build a green production mode with high scientific and technological content and low resource consumption and environmental pollution. In this context, the key to China’s industrial green transformation lies in coordinating economic development, resource conservation, and environmental protection [3]. Eco-efficiency is an important index to measure the coordinated development of the economy and ecological environment. It is not only a relatively comprehensive concept, but also includes various subcategories such as energy efficiency, resource efficiency, and environmental efficiency [4]. Its basic connotation is maximizing output and minimizing resource consumption and environmental damage to achieve sustainable development [5]. Therefore, improving eco-efficiency is the starting point for integrating China’s economic growth, resource conservation, and environmental protection. It plays a key role in achieving green development in China and the world.

As a spatial organization mode formed by the deepening of the industrial division of labor, industrial agglomeration concentrates enterprises of the same or different industries in a specific region, resulting in the formation of external economies of scale [6]. With the acceleration of China’s economic system reform, the agglomeration has become an inevitable trend and an important mode of industrial development [7]. Many industrial enterprises gather in the eastern coastal areas, forming various industrial clusters. The government has also established various special economic zones, such as industrial parks, technological innovation parks, and free trade zones. However, in the formation and evolution of industrial agglomeration, some cities have formed a specialized agglomeration model of “one industry dominates”, such as resource-based cities. Other cities have formed diversified agglomeration models of “small and comprehensive” or “large and all-inclusive”. Therefore, it is ambiguous which agglomeration mode brings the most substantial improvement in the eco-efficiency of a city.

Theoretically, industrial agglomeration may have both positive and negative effects on eco-efficiency. On the one hand, industrial agglomeration promotes labor productivity and economic growth through the positive effects of knowledge spillover and scale economies. In addition, industrial agglomeration reduces energy consumption and pollutant emissions through the spillover of clean and environmental protection technologies, the sharing of pollution control facilities, and the development of a circular economy [8]. On the other hand, the expansion of the production scale caused by industrial agglomeration increases energy consumption and pollutant emissions, which produces a crowding effect [7]. Overall, industrial agglomeration has a complex impact on eco-efficiency. As the three basic modes of industrial agglomeration, specialization, related diversified, and unrelated diversified have different action mechanisms on economic growth and environmental pollution owing to differences in organizational modes. Therefore, each agglomeration mode has a significantly different impact on eco-efficiency. Moreover, each agglomeration mode has different externalities of economy and environment (i.e., positive or negative) at various stages of agglomeration, resulting in a nonlinear relationship between each agglomeration mode and eco-efficiency. This relationship also depends on the stage of industrialization, market demand, resource conditions, and openness. As the dominant feature of a city, city size is the external manifestation of many restrictions to a great extent. Therefore, the heterogeneity of city size increases differences in the impact of industrial agglomeration on eco-efficiency. Based on this analysis, the research question of this paper focuses on how different agglomeration modes dynamically affect industrial eco-efficiency. Obviously, exploring this issue helps to clarify which agglomeration model is more conducive to the improvement of eco-efficiency, which deepens the research on the influencing mechanism of eco-efficiency. In addition, it is important for the realization of industrial green transformation and development.

The contribution of this paper mainly includes four aspects. Firstly, the paper distinguishes the impact mechanism of different agglomeration modes on eco-efficiency. Secondly, it calculates the specialized, related diversified, and unrelated diversified agglomeration index of Chinese cities using the micro-level data of the China Industrial Enterprise Database, and measures the industrial eco-efficiency of each city using the super-efficiency SBM-DEA model with undesirable outputs. Thirdly, it employs a panel threshold model to test the nonlinear impact of specialized agglomeration and related and unrelated diversified agglomeration on industrial eco-efficiency. This study also uses the static and dynamic spatial panel to examine the robustness of the nonlinear relationship. Finally, it discusses the impact of city-size heterogeneity on the relationship between the three agglomeration models and industrial eco-efficiency.

The rest of this paper is organized as follows. The next section is a literature review. The third section analyzes the influence path. The fourth section explains methodology, variables, and data sources. The fifth section gives the empirical results and discussion. Finally, the last section provides the conclusion and policy implications.

## 2. Literature Review

### 2.1. Industrial Agglomeration and Economic Growth

Since Marshall proposed the concept of industrial agglomeration, scholars have widely discussed the effects of industrial agglomeration on economic growth, labor productivity, and technical innovation. Most studies believe that industrial agglomeration produces scale economies and knowledge spillover effects, which promotes economic growth [9,10,11,12]. However, some studies show that industrial agglomeration inhibits economic growth due to the crowding effect [13,14]. In addition, many studies claim that the positive and negative externalities of industrial agglomeration follow a dynamic game process. The dominant externality determines the impact of industrial agglomeration on economic growth or labor productivity, forming a nonlinear relationship [15,16].

Furthermore, researchers divide industrial agglomeration into two modes of specialized and diversified agglomeration, according to whether the externality of agglomeration comes from the same industrial type. According to Marshall [17], the MAR externality generated by specialized agglomeration increases knowledge spillover among enterprises, which promotes the innovation and growth of the industry. Jacobs [18] believes that the Jacobs’ externality generated by diversified agglomeration is more conducive to promoting knowledge spillover and industrial growth. Although most studies believe that industrial agglomeration has an important impact on economic growth, they have fundamental disagreements about the effects of specialized and diversified agglomerations. Many scholars argue that diversified agglomeration increases economic growth. Glaeser et al. [19] show that diversified agglomeration increases industrial growth, while specialized agglomeration reduces industrial growth in the United States. Batisse’s research on China also confirms this finding [20]. Other researchers claim that the positive effect of specialized agglomeration on economic growth is greater than that of the diversified one. Henderson [21] and Klein and Crafts [22] show that the specialized agglomeration effect is more significant than the diversified one in promoting American manufacturing industry development. Zhu et al. [23] also suggest that specialized agglomeration is beneficial to economic development using data on Chinese cities, while diversified agglomeration hinders economic development to a certain extent. Other scholars find that both specialized and diversified agglomeration promote economic growth significantly. Forni and Paba [24], Blien et al. [25], and Simonen et al. [26] confirm this finding in their empirical studies on Germany, Finland, and Italy, respectively. In addition, many researchers accept the nonlinear relationship of specialized and diversified agglomerations with industrial growth. de Lucio et al. [27] affirm a U-shaped relationship between specialization and productivity growth in Spain, while diversification shows an insignificant impact on productivity growth. Hu and Sun [28] claim that specialization and diversification have a nonlinear relationship with economic development under the influence of innovation level and urban size in China.

Although many studies confirm Jacobs’ view, a large number of studies show that diversified agglomeration does not bring knowledge spillover or growth. In fact, two completely unrelated industries establish an ineffective knowledge spillover channel. Only industrial sectors with complementary elements and knowledge can effectively produce knowledge spillover and promote regional economic growth. Therefore, Frenken et al. [29] first divided industrial diversification into related and unrelated diversification, and then scholars began to carry out empirical research on the role of related and unrelated diversification in economic growth. Falcıoğlu [30], Boschma et al. [31], and Cainelli et al. [32] confirm that related diversification promotes economic growth or productivity improvement, using data on Turkey, Spain, and Italy, respectively.

### 2.2. Industrial Agglomeration and Environmental Pollution

As environmental pollution has become more and more prominent in recent years, numerous scholars have studied the impact of industrial agglomeration on environmental pollution. However, there is serious disagreement on the relationship between industrial agglomeration and environmental pollution. One view is that industrial agglomeration intensifies environmental pollution since the expansion of production scale caused by agglomeration increases pollutant emissions. Virkanen [33] believes that industrial agglomeration in southern Finland is an important factor causing air and water pollution. Dong et al. [34] and Cao et al. [35] argue that industrial agglomeration exacerbates pollutant emissions using China’s inter-provincial panel data. Sun and Yuan [36], Cheng [37], Liu et al. [38], Liu et al. [39], Li, Xu and Yao [7], and Lu et al. [40] also confirm this view using China’s urban-level data. Another view is that industrial agglomeration reduces environmental pollution via the spillover of green knowledge and technology and the sharing of environmental protection facilities and resource recycling systems. Zeng and Zhao [41] show that industrial agglomeration alleviates the effect of pollution havens. Wang et al. [42] suggest a negative correlation between industrial agglomeration and pollutant emissions in Foshan, China. Fang et al. [43] find that manufacturing agglomeration reduces the haze pollution of local cities and surrounding cities through the technology spillover effect in China. In addition to the above two views, other studies believe that there is an uncertain relationship between industrial agglomeration and environmental pollution since the impact of industrial agglomeration on the environment has both positive and negative externalities. Its overall impact depends on which externalities dominate in different stages of agglomeration. He et al. [44] accept an inverted U-shaped relationship between industrial agglomeration and industrial SO_2_ emissions, and Wang and Wang [45] confirm a U-shaped relationship between the industrial agglomeration of Chinese cities and the emission intensity of sulfur dioxide.

Despite the increasing number of studies on the impact of industrial agglomeration on environmental pollution, only a few studies discuss the impact of different agglomeration modes on environmental pollution. Deng and Xu [46] argue that both specialized and diversified agglomeration enhance the emission reduction effect of foreign direct investment, but there are differences in cities with various sizes. Xie and Yuan [47] used the panel threshold model to find a U-shaped relationship between specialized agglomeration and pollution, while the nexus of diversified agglomeration and pollution is complex. Han et al. [48] show that both specialized and diversified agglomeration have an inverted U-shaped impact on environmental pollution. In addition, few studies discuss the impacts of related and unrelated diversification on environmental pollution. Wang et al. [49] affirm that related diversification reduces industrial pollution emission intensity, while unrelated diversification aggravates pollution emissions. Hu et al. [50] also confirm that related diversification reduces industrial SO_2_ emission intensity, while the impact of unrelated diversification is obscure.

### 2.3. Industrial Agglomeration and Eco-Efficiency

Although there is a limited amount of research on the impact of industrial agglomeration on eco-efficiency, there are profound disagreements among their findings. Some studies show that industrial agglomeration improves eco-efficiency significantly. Otsuka et al. [51] found that industrial agglomeration improves energy efficiency in Japanese cities. Liu, Cheng and Zhang [8] also claim that industrial agglomeration has a significant positive impact on energy efficiency using statistical data on prefecture-level cities in China. Other scholars believe that there is a nonlinear relationship between industrial agglomeration and eco-efficiency. Zheng and Lin [52] use the provincial panel data of China’s paper industry to show that industrial agglomeration positively impacts energy efficiency only when the agglomeration passes a threshold. Zhao and Lin [53] support an inverted U-shaped relationship between industrial agglomeration and energy efficiency using the provincial panel data of China’s textile industry. Guo et al. [54] confirm a U-shaped relationship between industrial agglomeration and green development efficiency in Northeast China.

To sum up, there are relatively few studies on the impact of industrial agglomeration on eco-efficiency, and they mainly focus on the linear relationship between industrial agglomeration and eco-efficiency. The research on the nonlinear relationship between them is insufficient and limited to some industries or regions. More importantly, the existing research ineffectively distinguishes the impact of different agglomeration modes on eco-efficiency, especially the impact of related and unrelated diversified agglomeration. In addition, the current research ignores the role of heterogeneous factors, such as city size, in the impact of industrial agglomeration on eco-efficiency. 

## 3. Influence Path of Different Agglomeration Modes on Eco-Efficiency

### 3.1. Influence Path of Specialized Agglomeration on Eco-Efficiency

Specialized agglomeration improves innovation ability and reduces production costs and pollution emissions through the scale economies of labor, intermediate inputs and infrastructure, and the knowledge spillover effect. These factors promote the improvement of eco-efficiency. When a specific space gathers the specialized labor force, enterprises can easily obtain the required labor force according to their own needs, and the highly skilled workers in the region can also find jobs matching their skills, which reduces the labor search cost and improves labor productivity [55]. The intermediate input market provides specialized products and services for enterprises, reducing transaction costs and resource consumption in the transportation process and increasing economies of scale and eco-efficiency. Due to the high similarity of the needs of industrial enterprises in the same industry for infrastructure, specialized agglomeration paves the way for sharing pollution control facilities and other supporting facilities. In this way, it reduces the unit cost of pollution treatment through the scale effect [47,56]. Finally, specialized agglomeration can encourage enterprises to realize all kinds of explicit and tacit knowledge spillover through imitation, business interaction, and the flow of technicians among enterprises [57], especially the spillover of environmental protection and cleaning production knowledge [6,43]. At the same time, the spillover of this knowledge has also created conditions for “learning by doing” and “learning by using”. Employees can improve products and equipment in the process of using this knowledge to solve production problems, meet consumer needs, and overcome technical problems, which promotes green innovation and the improvement of eco-efficiency in the agglomeration area.

However, specialized agglomeration produces many negative externalities, which hinder the improvement of eco-efficiency. On the one hand, specialized agglomeration expands production capacity and aggravates energy consumption and pollutant emissions [37]. On the other hand, excessively specialized agglomeration produces a crowding effect, which increases factor resource prices and production costs, and puts great pressure on pollution control facilities. In this way, it decreases eco-efficiency [8]. In addition, specialized agglomeration produces innovation path dependence and cognitive self-locking, which restricts the improvement of innovation abilities.

### 3.2. Influence Path of Related Diversified Agglomeration on Eco-Efficiency

Related diversified agglomeration improves eco-efficiency through scale economies by sharing labor and infrastructure, the spillover effect of knowledge and technology, and the circular economy effect. Related diversified agglomeration provides labor with different skills, which reduces the search cost and improves the matching efficiency of the labor market. Furthermore, it helps enterprises to share all kinds of environmental protection infrastructure and promotes the formation of a specialized pollution control market, which finally produces the scale economies effect of environmental governance [1]. Due to the appropriate cognitive distance between industries, related diversified agglomeration stimulates effective interactive learning and innovation by promoting exchanges and cooperation among enterprises in relevant industries. It promotes the spillover of environmental protection technology and the improvement of the innovation level [58]. At the same time, the forced effect of competition among enterprises also continuously encourages enterprises to improve their technological innovation ability and eco-efficiency. From the circular economy perspective, related diversified agglomeration encourages affiliated enterprises to form a symbiotic relationship of material resource exchange. The by-products or wastes produced by an enterprise may be raw materials or intermediate inputs required by other enterprises. It recycles material resources in the agglomeration area and reduces pollution emissions [59]. In addition, the geographical agglomeration of complementary enterprises reduces energy consumption and product loss in the process of transportation. All of these factors significantly improve eco-efficiency. However, related diversified agglomeration produces a crowding effect (i.e., excessive agglomeration leads to excessive resource consumption and environmental pollution, followed by a decline in eco-efficiency).

### 3.3. Influence Path of Unrelated Diversified Agglomeration on Eco-Efficiency

As a mode of agglomeration without obvious economic and technological links, unrelated diversified agglomeration results in the limited sharing of labor and infrastructure such as pollution control facilities, which hinders the exertion of scale economies. Due to the lack of complementary factor inputs among enterprises in different industries, enterprises in industrial agglomeration areas lack input-output linkages, which is detrimental to the formation of the circular economy effect. In the short term, there are few opportunities for environmental protection knowledge and technology exchange among enterprises due to the great cognitive distance between enterprises. It limits the spillover of various progressive technologies. In the long run, however, unrelated diversified agglomeration achieves breakthrough innovation and improves eco-efficiency since the reorganization of unrelated technologies leads to the emergence of new operating principles, functions, and applications [60]. Moreover, the combination of unrelated industries avoids the transmission of external shocks to other industries through the industrial chain, which enhances the stability of economic development and reduces the volatility of eco-efficiency [29].

## 4. Methodology, Variable Description, and Data

### 4.1. Methodology

#### 4.1.1. The Super-Efficiency SBM-DEA Model with Undesirable Outputs

This paper adopts Data Envelopment Analysis (DEA) to measure the industrial eco-efficiency of Chinese cities. The DEA model treats each city as a Decision-Making Unit (DMU) and mainly uses linear programming technology to make a productive evaluation of the DMU. Compared with the parametric frontier approach, such as the stochastic frontier analysis (SFA), the DEA model not only does not need to provide prior weight information or set definite function form, but it can also easily deal with the efficiency measurement problem under the condition of multi-inputs and multi-outputs. Therefore, the DEA model has become an effective tool to evaluate eco-efficiency in various studies. However, the traditional DEA model, such as the CCR (Charnes, Cooper and Rhodes) and BCC (Banker, Charnes and Cooper) models, ignores the relaxation of input and output and ineffectively measures the efficiency with undesirable output. Therefore, Tone [61] proposed a DEA model based on a Slack-Based Measure (SBM) in 2001. The SBM-DEA evaluation model directly adds the relaxation variable to the objective function, solving the problem of input-output relaxation. Considering that the model also faces the problem that the efficiency values of effective DMUs cannot be further distinguished, Tone [62] proposed the super-efficiency SBM-DEA model in 2002. It can effectively compare and evaluate DMUs at the forefront owing to combining the advantages of super-efficiency DEA and the SBM-DEA model. Tone and Sahoo [63] further proposed the super-efficiency SBM-DEA model with undesirable outputs in 2004 in order to be consistent with the actual production process. Equation (1) shows the model.

(1) $$\begin{array}{c} \rho =\min\frac{1-(1/m)\sum_{i=1}^{m}\left(w_{i}^{-}/x_{it}\right)}{1+1/(r_{1}+r_{2})(\sum_{s=1}^{r_{1}}w_{s}^{d}/y_{st}^{d}+\sum_{q=1}^{r_{2}}w_{q}^{u}/y_{qt}^{u})}\\ x_{it}=\sum_{j=1}^{n}x_{ij}\lambda_{j}+w_{i}^{-}\;i=1,\ldots ,m\\ y_{st}^{d}=\sum_{j=1}^{n}y_{sj}^{d}\lambda_{j}-w_{s}^{d}\;s=1,\ldots ,r_{1}\\ y_{qt}^{u}=\sum_{j=1}^{n}y_{qj}^{u}\lambda_{j}+w_{q}^{u}\;q=1,\ldots ,r_{2}\\ \lambda_{j}>0\;j=1,\ldots ,n\\ w_{i}^{-}\geq 0\;i=1,\ldots ,m\\ w_{s}^{d}\geq 0\;s=1,\ldots ,r_{1}\\ w_{q}^{u}\geq 0\;q=1,\ldots ,r_{2} \end{array}$$

where *m*, *r*_1_, and *r*_2_ denote the number of input, desirable output, and undesirable output indicators, respectively; ${w_{i}}^{-}=0$ is the input slack variable; ${w_{s}}^{d}=0$ shows the desirable output slack variable; ${w_{q}}^{u}=0$ denotes the undesirable output slack variable; $x_{it}$ is the input value of city *j* in year t; $y_{st}^{d}$ represents the desirable output value of city *j* in year t; $y_{qt}^{u}$ shows the undesirable output value of city *j* in year *t*; $\lambda_{j}$ is the coefficient; and ρ denotes the industrial eco-efficiency of a city, and the greater the value, the higher the industrial eco-efficiency. The objective function ρ is a strictly decreasing function of $w_{i}^{-}$, $w_{s}^{d}$, and $w_{q}^{u}$, ranging between 0 and 1. For specific evaluation units, it is efficient if and only if ρ = 1 and $w_{i}^{-}=w_{s}^{d}=w_{q}^{u}=0$; otherwise, it is inefficient, and the input-output needs improvement.

#### 4.1.2. Threshold Regression Model Setting

This paper empirically tests the dynamic impact of industrial agglomeration on industrial eco-efficiency using the panel threshold model proposed by Hansen [64]. The panel threshold model analyzes the degree or direction of variable influence after reaching a certain threshold value. Compared with the ordinary panel regression model, the panel threshold model can more accurately fit the nonlinear relationship between explanatory and explained variables in different groups. The basic equation for the panel threshold model is as follows:

(2) $$Y_{it}=\alpha X_{it}+\beta_{1}E_{it}I(T_{it}\leq \lambda )+\beta_{2}E_{it}I(T_{it}>\lambda )+C+\varepsilon_{it}$$

where *i* and *t*, respectively, are individual and time; *Y_it_* is the explained variable; *E_it_* shows the core explanatory variable; *T_it_* denotes the threshold variable; λ represents the threshold value as a parameter; *X_it_* is the control variable; α shows the coefficient vector corresponding to *X_it_*; $\beta_{1}$ and $\beta_{2}$ are the coefficients which should be estimated for each variable; C is the constant term; $\varepsilon_{it}$ represents the disturbing term; and *I(·)* is the characteristic function, which is 1 when the conditions in the parentheses are met and 0 when they are not fulfilled. To generate an estimate of the parameters, we first need to deduct the group average from the observed values of each variable in Equation (2), to transform it into:

(3) $$Y_{it}^{*}=\alpha X_{it}^{*}+\beta_{1}E_{it}^{*}I(T_{it}\leq \lambda )+\beta_{2}E_{it}^{*}I(T_{it}>\lambda )+C+\varepsilon_{it}^{*}$$

We use the Ordinary Least Square (OLS) method to estimate $\beta_{1}$ and $\beta_{2}$, and calculate the corresponding sum of squared errors S(λ)=e(λ)′e(λ). Minimizing the sum of the squared errors easily determines threshold estimators, that is, $\hat{\lambda }=\arg\min S(\lambda )$.

Then, we test the threshold effect and estimate. First, we check the significance of the threshold effect. The linear constraint *H_0_:* $\beta_{1}$($\hat{\lambda }$) = $\beta_{2}$($\hat{\lambda }$) represents the null hypothesis. The Likelihood Ratio (LR) test statistic is:

(4) $$F=(S_{0}-S(\hat{\lambda }))/{\hat{\sigma }}^{2}$$

where $S_{0}$ is the sum of squares of errors obtained by the constraint of the null hypothesis. ${\hat{\sigma }}^{2}$ is the corresponding variance of the errors in the alternative hypothesis. The threshold value λ is uncertain in the null hypothesis, and hence the F statistic follows a non-standard distribution. Hansen proposes a bootstrap method to produce the first-order asymptotic distribution for estimating the corresponding *p*-value.

The next step is to test the authenticity of the threshold estimate. The null hypothesis *H_0_：* $\hat{\lambda }$ = λ′ shows that the threshold estimators are equal to the actual values. The corresponding LR test statistic is as follows:

(5) $$LR_{1}=\frac{S_{1}(\lambda \prime )-S_{1}(\hat{\lambda })}{{\hat{\sigma }}^{2}}$$

The distribution of the statistic $LR_{1}(\lambda \prime )$ is also non-standard. To this end, Hansen constructed a function: $c(\alpha )=-2\ln(1-\sqrt{1-\alpha )}$. If $LR_{1}$ ≤ c(α), the null hypothesis is rejected where α is the statistical significance level.

This research conducts the above parameter estimation and hypothesis test methods regarding the single threshold model. The multi-threshold model is an extension of the single threshold model [64].

According to Equation (2), this paper takes the indicators of specialized, related diversified, and unrelated diversified agglomeration as threshold variables to study the impacts of different industrial agglomeration models on industrial eco-efficiency. Taking the existence of a threshold as an example, the model is set as follows:

(6) $$Iee_{it}=\alpha X_{it}+\beta_{1}Spe_{it}I(Spe_{it}\leq \lambda )+\beta_{2}Spe_{it}I(Spe_{it}>\lambda )+C+\varepsilon_{it}$$

(7) $$Iee_{it}=\alpha X_{it}+\beta_{1}Rd_{it}I(Rd_{it}\leq \lambda )+\beta_{2}Rd_{it}I(Rv_{it}>\lambda )+C+\varepsilon_{it}$$

(8) $$Iee_{it}=\alpha X_{it}+\beta_{1}Ud_{it}I(Ud_{it}\leq \lambda )+\beta_{2}Ud_{it}I(Ud_{it}>\lambda )+C+\varepsilon_{it}$$

where *Iee_it_* represents industrial eco-efficiency; *Spe_it_* is specialized agglomeration; *Rd_it_* and *Ud_it_* are related and unrelated diversified agglomerations, respectively; and *X_it_* represents a group of control variables.

Then, this paper investigates if city size affects the relationship between industrial agglomeration and eco-efficiency. To this end, our research constructs a panel threshold model with the city size (city population) as the threshold variable to explore the impact of city-size heterogeneity on the relationship between different agglomeration models and industrial eco-efficiency. Taking a single threshold as an example, the model is as follows:

(9) $$Iee_{it}=\alpha X_{it}+\beta_{1}Spe_{it}I(Us_{it}\leq \lambda )+\beta_{2}Spe_{it}I(Us_{it}>\lambda )+C+\varepsilon_{it}$$

(10) $$Iee_{it}=\alpha X_{it}+\beta_{1}Rd_{it}I(Us_{it}\leq \lambda )+\beta_{2}Rd_{it}I(Us_{it}>\lambda )+C+\varepsilon_{it}$$

(11) $$Iee_{it}=\alpha X_{it}+\beta_{1}Ud_{it}I(Us_{it}\leq \lambda )+\beta_{2}Ud_{it}I(Us_{it}>\lambda )+C+\varepsilon_{it}$$

where *Us_it_* represents city size.

### 4.2. Variable Description

#### 4.2.1. Explained Variable: Industrial Eco-Efficiency (Iee)

The determination of input, desirable output, and undesirable output variables is necessary to measure the industrial eco-efficiency of cities using the super-efficiency SBM-DEA model. The relevant indicators are as follows. (1) Capital input: the net fixed assets of industrial enterprises above designated size in each city as a proxy for capital investment. (2) Labor input: the annual average number of employees in industrial enterprises above the designated size in each city as the labor input indicator. (3) Energy input: the annual industrial electricity consumption of each city. (4) Desirable output: the gross industrial output value of industrial enterprises above the designated size in each city. (5) Undesirable output: the total volumes of industrial wastewater, industrial SO_2,_ and industrial soot (dust) in each city.

#### 4.2.2. Core Explanatory Variable: Industrial Agglomeration

(1) Specialization (Spe)

Previous studies employed the Location Quotient (*LQ*), Concentration Ratio (*CR*), and Krugman Specialization Index (*KSI*) to measure the specialization level of industries. Considering our data and research purpose, we use *KSI* to measure the specialized level of the urban industry. The calculation formula for *KSI* is as follows:

(12) $$KSI=\sum_{j=1}^{n}\left|\frac{s_{ij}}{s_{i}}-\frac{s_{j}}{s}\right|$$

where *i* is the city; *j* is the industrial sector; *S_ij_* denotes the output value of *j* industrial sector in city *i*; *S_i_* and *S_j_* denote the gross output value of the industry in city *i* and industrial sector *j* in all sample cities, respectively; and *S* denotes the gross output value of the industry in all sample cities. *KSI* ranges within 0–2, and the greater the value of *KSI*, the higher the level of specialized agglomeration in a city.

(2) Related diversification (Rd) and Unrelated diversification (Ud)

Following Frenken, Van Oort and Verburg [29], this paper uses the entropy index method to measure the degree of related and unrelated diversified agglomerations. Entropy refers to the degree of chaos in a system. The entropy index method captures variety by measuring the ‘uncertainty’ of probability distributions. The main advantage of this method is that entropy can be decomposed at each sectoral digit level. The decomposable nature of entropy implies that variety at several digit levels can enter a regression analysis without necessarily causing collinearity. Equation (13) represents industrial diversification according to the method of entropy index.

(13) $$Div=\sum_{j=1}^{n}P_{j}\ln(1/P_{j})$$

where *P_j_* represents the output proportion of industrial sector *j* to the gross output value of the industry in a city.

Assumedly, the industrial sector *j* is small, and all the small industrial sectors E_j_ (j = 1,…, n) can be aggregated into a smaller number of large industrial sectors S_1_,…, S_G_ in such a way that each small industrial sector falls exclusively under a large industrial sector S_g_ (g = 1,…, G). The share of the large industrial sector *P_g_* is the summation of the share of small industrial sectors *P_j_*: $P_{g}=\sum_{j\in g}P_{j}$. Equation (14) represents the diversification degree of various small industrial sectors within a large industrial sector.

(14) $$H_{g}=\sum_{j\in g}(P_{j}/P_{g})\ln(P_{g}/P_{j})$$

Finally, the industrial diversification index of the city is decomposed as follows:

(15) $$\begin{array}{l} Div=\sum_{j=1}^{n}P_{j}\ln(1/P_{j})=\sum_{g=1}^{G}\sum_{j\in g}P_{j}\ln(1/P_{j})\\ =\sum_{g=1}^{G}\sum_{j\in g}P_{j}[\ln(P_{g}/P_{j})+\ln(1/P_{g})]\\ =\sum_{g=1}^{G}[\sum_{j\in g}P_{g}(P_{j}/P_{g})\ln(P_{g}/P_{j})]+\sum_{g=1}^{G}[\sum_{j\in g}P_{j}\ln(1/P_{g})]\\ =\sum_{g=1}^{G}P_{g}[\sum_{j\in g}\left(P_{j}/P_{g}\right)\ln(P_{g}/P_{j})]+\sum_{g=1}^{G}P_{g}\ln(1/P_{g})\\ =\sum_{g=1}^{G}P_{g}H_{g}+\sum_{g=1}^{G}P_{g}\ln(1/P_{g})=Rd+Ud \end{array}$$

where $Rd=\sum_{g=1}^{G}P_{g}H_{g}$ is the weighted sum of entropy within each large industrial sector. The greater the weighted sum of entropy, the higher the degree of related diversified agglomeration. $Ud=\sum_{g=1}^{G}P_{g}\ln(1/P_{g})$ is the entropy at the large industrial sector level. The greater the entropy, the higher the degree of unrelated diversified agglomeration.

Based on the output value data of industrial enterprises above designated size in the China Industrial Enterprise Database, this paper calculates the specialized, related diversified, and unrelated diversified agglomeration indexes of city industry. Following Lin’s [65] method, this paper excludes the enterprise samples with an industrial output value of 0, less than 10 employees, and limited accounting standards. Moreover, this paper extracts the output value of 39 two-digit industrial sectors and 191 three-digit industrial sectors of each prefecture-level and higher-level city from mining, manufacturing, production, and supply of electricity, gas, and water, according to the industrial classification of the national economy (GB/T4754-2002). Then, this study summarizes the gross output value of the industry in each city and calculates the specialized agglomeration index based on the output value of two-digit industrial sectors in each city. Due to the large technical differences and weak correlation among the two-digit industrial sectors and the strong technical complementarity and substitution among the three-digit industrial sectors, this paper takes the two- and three-digit industrial sectors as the large and small industrial sectors, respectively, to calculate the related and unrelated diversified agglomeration indexes of each city.

#### 4.2.3. Control Variables

(1) Environmental Regulation (Er)

Strict and appropriate environmental regulation reduces the scale of polluting industries and enhances the innovation of environmental protection technology in enterprises, which reduces the intensity of pollution emissions and improves eco-efficiency. Using the entropy method, the paper selects the removal rate of industrial soot (dust) and SO_2_ and the treatment rate of industrial wastewater of each city to construct the comprehensive index of environmental regulation.

(2) Industrial structure (Is)

The increasing proportion of pollution-intensive industries expands the scale and intensity of pollution emissions, which affects eco-efficiency adversely. This paper defines pollution-intensive industries according to Guanghui et al. [66] and expresses the industrial structure by the proportion of the output value of pollution-intensive industries in the total industrial output value.

(3) Technology innovation (Tech)

Technology innovation is not only an important driving force of economic growth, but also an important way to reduce environmental pollution and improve resource utilization efficiency, which has a positive impact on eco-efficiency. Considering that invention patents can objectively reflect the original innovation capacity of a region or enterprise, this paper uses the authorization amount of invention and utility model patents to reflect technology innovation capacity.

(4) Foreign direct investment (Fdi)

Foreign direct investment provides advanced environmental protection technology for local enterprises, which improves industrial eco-efficiency. However, foreign direct investment causes environmental pollution due to the pollution haven effect [67], lowering industrial eco-efficiency. This paper uses the proportion of foreign direct investment to Gross Domestic Product (GDP) to characterize the degree of foreign direct investment.

(5) Economic development level (Edl)

Various modes of social production at different levels of economic development have heterogeneous levels of resource utilization, which have an important impact on industrial eco-efficiency [53]. Per capita GDP measures the economic development level of a city.

(6) Government intervention (Gi)

The government’s limited intervention in the market effectively alleviates the problems of information asymmetry and monopoly caused by market failure, which optimizes the allocation of resources and improves industrial eco-efficiency. However, if the government intervenes excessively, it leads to the inefficiency of resource allocation and reduces industrial eco-efficiency. Therefore, this paper uses the proportion of the city’s fiscal expenditure to GDP to measure the degree of government intervention.

(7) Other

It should also be noted that specialized, related diversified, and unrelated diversified agglomeration indexes are also alternately regarded as control variables.

### 4.3. Data

Considering the adjustment of administrative division and data accessibility, this paper selects 283 prefecture-level and higher-level cities in China except for Chaohu, Bijie, Tongren, Lhasa, Longnan, and Zhongwei as the samples. Because the current data from the China Industrial Enterprise Database is only updated to the year 2013, and the standard of the national economic industrial classification has been significantly adjusted in 2003 and 2012, this paper sets the research period from 2004 to 2012 to ensure the consistency of the data. This period plays a key role in transforming China’s industrialization from accelerated development to a mature stage. In this period, not only did industrial agglomeration develop rapidly, but also the problem of resources and environment became prominent. Therefore, research on the relationship between industrial agglomeration and eco-efficiency is typical in this period. The data in this paper are mainly derived from the China Industrial Enterprise Database, China Urban Statistical Yearbook, and China regional economic statistical yearbook. The patent data come from the patent retrieval database of the State Intellectual Property Office (SIPO). To eliminate the impact of inflation, the gross industrial output value and per capita GDP have been adjusted into comparable prices according to the producer price index of industrial products in each province, and the net value of fixed assets have been adjusted into comparable prices according to the price index of fixed asset investment in each province. The base year is 2004 for all the variables, and the price indexes come from the China Statistical Yearbooks 2005–2013. Finally, the descriptive statistics show that the numerical distribution of each variable presents a relatively stable trend in Table 1.

## 5. Empirical Research and Discussion

### 5.1. Estimation Results of Industrial Eco-Efficiency

To highlight the characteristics of industrial eco-efficiency, this paper analyzes the industrial eco-efficiency from two aspects: the whole country and cities of different sizes. According to the standard of city size classification, Chinese cities are divided into four categories: super-large and mega-cities with a population of more than 5 million, large cities with a population of 1–5 million, medium-sized cities with a population of 0.5–1 million, and small cities with a population of less than 0.5 million. 

Firstly, from the perspective of nationwide characteristics (see Table 2), the average value of urban industrial eco-efficiency generally shows a steady upward trend, from 0.589 in 2004 to 0.704 in 2012, indicating that the transformation of urban industry from an extensive to intensive development mode has made some progress. Secondly, from the perspective of cities of different sizes (see Table 2), the overall average industrial eco-efficiency of small, medium, large, and mega-cities increased successively from 2004 to 2012, which were 0.609, 0.627, 0.730, 0.848, and 0.971, respectively. It indicates that industrial eco-efficiency continued to improve with the increase of city size. At the same time, the industrial eco-efficiency of cities of different sizes shows a fluctuant upward trend, but the rising range is different (see Table 2). Among them, the average industrial ecological efficiency in medium-sized cities is the largest, which is 21%; the average industrial eco-efficiency in mega-cities is the smallest, with an increase of only 3.47%; the average industrial eco-efficiency in small, large, and mega-cities are 16.9%, 16%, and 13.1%, respectively. The reason may be that the increasingly prominent economies of scale and continuously optimized resource allocation efficiency promote the continuous improvement of industrial eco-efficiency as the city size increases. However, when city size is too large, the negative externalities such as high cost of production factors and serious environmental pollution slow down the improvement of industrial eco-efficiency.

### 5.2. The Influence of Different Agglomeration Modes on Industrial Eco-Efficiency

This paper tests the threshold effect and estimates the threshold value of three models with specialized, related diversified, and unrelated diversification agglomeration as explanatory variables and threshold variables, respectively. It shows that the relationship between different agglomeration modes and industrial eco-efficiency is linear or nonlinear. This research tests the single-, double-, and triple-threshold of each model and repeats the bootstrap 300 times to obtain the LR F-statistic, the Bootstrap *p*-value, and critical values of the three panel threshold models (see Table 3). The LR F-statistic in the single or double threshold model of the specialized agglomeration rejects the null hypothesis at 1% statistical significance level, and the corresponding threshold values are 1.493, 1.493, and 1.632, respectively, but the triple threshold model shows an insignificant effect. The LR F-statistic of the single and double threshold models in the related diversified agglomeration reject the null hypothesis at 1% and 5% statistical significance levels, respectively, with threshold values of 0.179 and 0.376, while the triple threshold model shows an insignificant effect. The LR F-statistic of the single threshold model in the unrelated diversified agglomeration rejects the null hypothesis at 5% statistical significance level, and the threshold value is 2.512, while the double and triple threshold models have insignificance effects. Thus, there is a nonlinear relationship between different modes of industrial agglomeration and industrial eco-efficiency.

Column (1) of Table 4 shows the regression results of the panel threshold model when specialized agglomeration is the threshold and core explanatory variable. In this regression, when the specialized agglomeration is lower than the first threshold value of 1.493, its influence coefficient on industrial eco-efficiency is −0.086 and statistically significant at 1%. When the specialized agglomeration is between 1.493 and 1.632, its influence coefficient on industrial eco-efficiency is −0.028, which is statistically significant at 5%. When the specialized agglomeration exceeds the second threshold value of 1.632, its influence coefficient on industrial eco-efficiency changes to 0.029, which is statistically significant at 5%. These results show a U-shaped relationship between specialized agglomeration and industrial eco-efficiency. As the specialized agglomeration increases, its effect on industrial eco-efficiency shifts from negative to positive. The reason for the above results may be that the specialized agglomeration has entered a rapid development stage after China’s membership to the World Trade Organization in 2000. The rapid expansion of the agglomeration scale has significantly increased production capacity, resource consumption, and pollutant emissions. At this stage, the knowledge and technology spillover effect is insignificant among enterprises. In case of weak environmental regulation, enterprises also lack external supervision pressure and an internal driving force for the research and application of environmental protection technology. Due to the insufficient development of infrastructure (for example, environmental protection facilities), the scale economies of specialized agglomeration play an inactive role in pollution control. In addition, the blind competition of enterprises in the same industry results in redundant construction and energy waste. All these factors decrease industrial eco-efficiency. When the specialized agglomeration reaches a certain degree, the effect of scale economies caused by the specialized agglomeration becomes increasingly prominent, resulting in a significant decrease in the resource consumption per unit of output. Furthermore, the specialized agglomeration promotes the spillover of environmental protection knowledge, which effectively reduces pollution emissions. In addition, the production activities of the same industry within the specialized agglomeration area typically generate the same or similar pollutants, making it easier to realize the scale effect of pollution control. On the whole, the specialized agglomeration significantly increases industrial eco-efficiency at this stage.

Column (2) of Table 4 presents the regression results of the panel threshold model when the related diversification is both the threshold variable and the core explanatory variable. Based on the results, when the related diversified agglomeration is less than the first threshold value of 0.179, its influence coefficient on industrial eco-efficiency is −0.026, which is statistically significant at 5%. When the related diversified agglomeration exceeds the first threshold value and ranges within 0.179–0.376, its influence coefficient on industrial eco-efficiency changes to 0.029 and is statistically significant at 5%. When the related diversified agglomeration exceeds the second threshold value of 0.376, the coefficient on industrial eco-efficiency further increases to 0.098, which is statistically significant at 5%. These results show a U-shaped relationship between related diversified agglomeration and industrial eco-efficiency. As related diversified agglomeration increases, its influence on industrial eco-efficiency shifts from negative to positive. The reason is that the related diversified agglomeration is still in the primary stage after 2000. Hence, there is a lack of an effective division of labor and cooperation among enterprises in the agglomeration area, leading to inefficient resource allocation and the discouragement of recycling pollutants and wastes. In addition, related diversified agglomeration limits the knowledge spillover effect and the innovation of environmental protection technology. Moreover, it is difficult to achieve the scale effect of pollution control due to the small agglomeration scale. Therefore, the related diversified agglomeration decreases industrial eco-efficiency at this stage. As the related diversified agglomeration increases, the division of labor and cooperation improves among enterprises, and some regions even encourage the recycling of material resources, which heightens resource utilization efficiency and reduces pollution emissions. Furthermore, sharing technology and information caused by related diversified agglomeration is also more frequent, especially the spillover effect of environmental protection knowledge which is increasingly prominent. In addition, the market of various production factors (such as labor force) is also increasingly perfect, reducing enterprise costs and optimizing resource allocation. These factors significantly improve urban industrial eco-efficiency.

Column (3) of Table 4 shows the regression results of the panel threshold model when the unrelated diversified agglomeration is both the threshold variable and the core explanatory variable. In the regression results, when the unrelated diversified agglomeration is lower than the threshold value of 2.512, its influence coefficient on industrial eco-efficiency is −0.082 and statistically significant at 1%. When the unrelated diversified agglomeration exceeds this threshold value, the coefficient changes to −0.055, which is statistically significant at 5%. These results show that unrelated diversified agglomeration decreases industrial eco-efficiency, but the negative impact weakens as the agglomeration degree increases. The reason is that it is difficult for enterprises to overflow knowledge and technology, and enterprises also lack the competitive pressure and motivation to develop environmental protection technologies due to the long distance of technology cognition among industries and the low correlation between input and output. Moreover, the level of labor division and cooperation among industries is low, and it is difficult to improve resource utilization efficiency. In addition, the sharing level of labor, intermediate inputs, and pollution control facilities are low among enterprises in different industries due to the differences in production factors required and the waste discharged. It increases the search and transaction costs of enterprises, making it difficult to achieve scale economies. These factors discourage industrial eco-efficiency. However, as the unrelated diversified agglomeration reaches a certain degree, the agglomeration of all kinds of heterogeneous knowledge and technology may also produce breakthrough innovation, reducing the negative impact of unrelated diversified agglomeration on industrial eco-efficiency.

Based on columns (1), (2), and (3) of Table 4, the coefficient of environmental regulation shows a significantly negative effect on industrial eco-efficiency, which is inconsistent with the expectation. Possible reasons are as follows. On the one hand, the implementation of environmental regulation is unsuccessful in stopping illegal emissions by enterprises. On the other hand, environmental regulation is mainly a command-control type, which has a limited incentive for green technology innovation leading to a reduction of industrial eco-efficiency. The industrial structure shows a significantly negative effect on industrial eco-efficiency, which is consistent with the expected results. It indicates that an increase in the proportion of polluting industries significantly decreases industrial eco-efficiency. Foreign direct investment shows a significantly negative effect on industrial eco-efficiency due to the pollution haven effect of China as a developing country. Technology innovation has a significantly positive effect on industrial eco-efficiency, which is consistent with the theoretical expectations. The economic development level has a significantly positive impact on industrial eco-efficiency, which suggests that the government and residents pay more and more attention to the environment as economic development increases, thus reducing environmental pollution. Government intervention has an insignificantly positive correlation with industrial eco-efficiency.

### 5.3. Robustness Test

The spatial correlation gradually increases among cities in the case of the free and frequent flow of production factors. Various economic and environmental activities of a city have spatial spillover effects on other cities. Therefore, variables (such as industrial agglomeration and eco-efficiency) have a strong spatial correlation. Based on this analysis, our paper uses a spatial panel model for the robustness test. The commonly used spatial weight matrices employ geographic adjacency, geographic distance, or economic distance matrices. Since the spatial correlation among regions comes from both the aspects of geography and economy, this paper constructs a comprehensive spatial weight matrix to integrate geographical and economic distance based on Xinshuo et al. [68] method.

Following Elhorst [69], this paper uses a non-spatial panel model to construct the Lagrange Multiplier (LM) and the Robust-Lagrange Multiplier (R-LM) for spatial correlation tests. As shown in Table 5, the LM-lag and LM-err as well as the R-LM-lag and R-LM-err of static and dynamic spatial panel models are statistically significant at 1%, confirming the selection of a spatial panel model. In addition, Hausman test results accept the fixed effects model. Then, we employ the LR and Wald tests to judge whether the spatial panel Durbin model (SPDM) can be simplified to the spatial panel lag model (SPAR) or spatial panel error model (SPEM). In Table 4, LR-sar, LR-sem, Wald-sar, and Wald-sem refuse the null hypothesis at a 5% significance level, indicating that the SPDM is the optimal spatial panel model. Therefore, this paper selects the static and dynamic SPDM with a fixed effect for the robustness test.

According to Table 6, the secondary coefficients of specialized agglomeration and related diversified agglomeration in the static and dynamic SPDM are positive in sign and statistically significant at 1%. Among them, the values of the specialized agglomeration at the inflection point are 1.677 and 0.761, respectively, which are within the sample value range (0.447–1.818). The values of the related diversified agglomeration at the inflection point are 0.232 and 0.597, respectively, which are also within the sample value range (0.447–1.818). This shows that the impact of specialized and related diversified agglomeration on industrial eco-efficiency presents a U-shaped feature. That is, the specialized and related diversified agglomeration firstly bring adverse effects on industrial eco-efficiency, but they then increase industrial eco-efficiency after exceeding the inflection point. The secondary coefficients of unrelated diversified agglomeration in the static and dynamic SPDM are negative and statistically significant at 5% and 1%, respectively. The values of the unrelated diversified agglomeration at the inflection point are −0.158 and 0.24, respectively, but the values of the two turning points are less than the sample value range (0.25–3.268) of the unrelated diversified agglomeration. This indicates that the unrelated diversified agglomeration reduces industrial eco-efficiency. The robustness test results are consistent with those of the panel threshold model, confirming the reliability of our results.

### 5.4. The Heterogeneity Effect of City Size

This paper explores the heterogeneity impact of city size on the relationship between different agglomeration modes and industrial eco-efficiency. To this end, it takes city population as the threshold variable and tests the models with specialized, related diversified, and unrelated diversified agglomeration as the core explanatory variables. As shown in Table 7, when specialized and related diversified agglomerations are the core explanatory variables, their single threshold models of city size reject the null hypothesis at 1%, while their double and triple thresholds are statistically insignificant. When unrelated diversified agglomeration is the core explanatory variable, the single threshold model of city size rejects the null hypothesis at 5%, and the double threshold model of city size rejects the null hypothesis at 1%, while the triple threshold model is statistically insignificant. Therefore, the model with specialized and related diversified agglomerations as explanatory variables adopts the single threshold model with the corresponding thresholds of 0.799 and 3.822 million persons, respectively. The model with unrelated diversified agglomeration as the threshold variable adopts the double threshold model with the thresholds of 1.491 and 4.971 million persons, respectively.

Based on the threshold value, our research estimates the above panel threshold model. Column (1) of Table 8 shows the regression results of the panel threshold model with the specialized agglomeration as the core explanatory variable. If the city size is less than the threshold of 0.799 million persons, the influence of the specialized agglomeration on industrial eco-efficiency is −0.014 and is statistically insignificant. If the city size exceeds 0.799 million persons, the influence of specialized agglomeration on industrial eco-efficiency changes to −0.061, which is statistically significant at 5%. These results show that when the city is below a specific size, the impact of specialized agglomeration on industrial eco-efficiency is insignificant. The specialized agglomeration has a significant inhibitory effect on industrial eco-efficiency when the city surpasses this size (i.e., medium-sized cities). The reason may be that the low cost of production factors (such as land and labor) meet the standard low production cost of specialized agglomeration in small cities, but small cities also limit the positive externalities (such as the sharing of supporting industries and infrastructure). Furthermore, small cities often involve industrial agglomeration with low technology and high energy consumption and pollution, resulting in serious environmental pollution. Therefore, the negative externalities of specialized agglomeration in small cities neutralize the positive ones, resulting in the insignificant impact of specialized agglomeration on industrial eco-efficiency. As the city size grows, the costs of various production factors such as land rent and labor force increase significantly. At the same time, the expansion of the agglomeration scale causes a shortage of resources and aggravates environmental pollution. Thus, the inhibitory effect of specialized agglomeration on industrial eco-efficiency gradually increases.

Column (2) of Table 8 shows the regression results of the panel threshold model with the related diversified agglomeration as the core explanatory variable. If the city size is less than 3.822 million persons, the influence of related diversified agglomeration on industrial eco-efficiency is 0.078 and is statistically significant at 1%. If the city size exceeds 3.822 million persons, the influence of related diversified agglomeration on industrial eco-efficiency increases to 0.134, which is statistically significant at 1%. This shows that the influence of related diversified agglomeration on industrial eco-efficiency has a significantly positive effect, enhanced further with the increase of city size. As the city size increases, the supply of various production factors, such as labor and intermediate inputs, improves the efficiency of enterprise resource allocation. Moreover, diversified infrastructure and public services help to form economies of scale, such as pollution control. At the same time, residents’ demands for environmental improvement increases, resulting in the continuous enhancement of environmental regulation. In this environment, cities gather low-pollution and high-tech industries and eliminate highly pollutant industries. These reasons lead to the positive externalities of related diversified agglomeration exceeding its negative externalities, which improves urban industrial eco-efficiency.

Column (3) of Table 8 shows the regression results of the panel threshold model with the unrelated diversified agglomeration as the core explanatory variable. If the city size is less than 1.491 million, the influence of unrelated diversification agglomeration on industrial eco-efficiency is −0.023 and is statistically significant at 5%. If the city size ranges between 1.491 and 4.972 million persons, the negative effect of unrelated diversified agglomeration on industrial eco-efficiency diminishes to −0.011, which is statistically significant at 10%. However, if the city size exceeds 4.972 million persons, the influence of unrelated diversified agglomeration on industrial eco-efficiency changes to 0.015, which is statistically significant at 10%. These results show that as the city size increases, the influence of unrelated diversified agglomeration on industrial eco-efficiency gradually changes from negative to positive, but the positive effect of unrelated diversified agglomeration appears only after reaching the size of super-large and mega-cities. As the city size increases, its capacity expands to accommodate more unrelated industries due to the increase in the variety of production inputs and outputs, market scale, and the improvement of infrastructure such as pollution control facilities. It alleviates the negative external effects caused by the increase of unrelated diversified agglomeration to a certain extent. Moreover, the increasing agglomeration of information, knowledge, and technology paves the way for enterprises to produce breakthrough innovations. In this environment, the increasing environmental regulation encourages low pollution and energy consumption and high technology in industries as the city size increases. These factors finally lead to the related diversified agglomeration promoting industrial eco-efficiency in super-large and mega-cities.

## 6. Conclusions and Policy Implications

The conclusions of this paper are as follows:

(1) The results show a nonlinear relationship between the three industrial agglomeration modes and industrial eco-efficiency. The impact of specialized agglomeration and related diversified agglomeration on industrial eco-efficiency presents a U-shape pattern of first hindrance and then promotion. The unrelated diversified agglomeration has a significantly negative impact on industrial eco-efficiency, but it weakens when agglomeration increases to a certain level. 

(2) From the perspective of city-size heterogeneity, if the city size is smaller than the threshold of 0.799 million persons, the impact of specialized agglomeration on industrial eco-efficiency is insignificant. However, the specialized agglomeration has a significant inhibitory effect on industrial eco-efficiency when the city size crosses this threshold, that is, after the city surpasses a medium size. The impact of related diversified agglomeration on industrial eco-efficiency increases as the city size grows. Moreover, when the city size reaches 3.822 million people, the promoting role of related diversified agglomeration is further enhanced. The impact of unrelated diversified agglomeration on industrial eco-efficiency gradually changes from negative to positive as the city size increases. However, its positive effect appears only in the super-large and mega-cities. Therefore, it is only the related diversified agglomeration that improves industrial eco-efficiency in cities of different sizes. Only the super-large and mega-cities benefit from unrelated diversification agglomeration, while improving industrial eco-efficiency in smaller cities does not necessarily benefit from specialized agglomeration.

Based on the above conclusions, this paper proposes the following policy implications:

(1) The relationship between different agglomeration modes and industrial eco-efficiency should be rapidly adjusted according to the dynamic change of agglomeration level. The impact of specialized agglomeration and related diversified agglomeration on industrial eco-efficiency is first suppressed and then promoted. Therefore, the government should promote the specialized agglomeration and related diversified agglomeration to cross the inflection point by adopting the market economy and related regulatory policies for the cities with less than the inflection point. In addition, the government should formulate relevant measures such as appropriate environmental regulations to avoid the phenomenon of “pollution first, treatment later”. For cities that have already passed the inflection points, the government should encourage technology innovation and optimize the industrial structure. It transforms the development of clustered industries into high-quality development with high income and low energy consumption and pollution. Moreover, the government should also appropriately control the level of agglomeration to avoid the negative externalities caused by excessive agglomeration. Due to the inhibitory effect of unrelated diversified agglomeration on industrial eco-efficiency, the government should cultivate leading industries according to its advantages and promote the vertical or horizontal extension of the industrial chain, which improves the correlation level among industries. Moreover, the government should strengthen the intensity of environmental regulation to eliminate backward production capacity and force the innovation of cleaner production technology.

(2) Differentiated policies of agglomeration should be adopted according to the size of cities. Small and some medium-sized cities should improve infrastructure and supporting services, such as environmental protection, and carry out technical training for workers to increase the supply of specialized labor. Based on the comparative advantages, such as resource endowment and the local industrial base, small and some medium-sized cities should concentrate limited resources on developing the key industries in a region. In addition, they should actively extend a clean industrial chain around the leading industries to improve industrial eco-efficiency. Large cities should encourage the coordinated development of multiple industries with considerable relevance and promote the transformation of clustered industries into high value-added and green industries. Due to the large urban capacity, super-large and mega-cities can also reduce the impact of economic fluctuations and promote breakthrough innovation by developing industries with low correlation, which improves industrial eco-efficiency.

Due to the availability of data, this paper only uses data from 2004 to 2012. Extending the research period to recent years will help to enhance the robustness of our research results and provide more enlightenment for the green transformation of China’s industry. This can be left as a possible issue for future research.

## Figures and Tables

**Table 1:** Descriptive statistics.

Variables	Min	Max	Mean	Std. Dev.
Iee	0.18	1.52	0.671	0.185
Spe	0.447	1.818	1.034	0.267
Rd	0.013	1.397	0.678	0.288
Ud	0.25	3.268	2.362	0.518
Er	0.006	5.327	0.976	0.767
Is	0.073	1	0.668	0.212
Tec	1	34,382	691.151	2114.288
Fdi	0	0.376	0.022	0.026
Edl	1860.017	128,931.9	18,691.84	15,792.52
Gi	0.031	0.885	0.139	0.074

**Table 2:** Evolution of average industrial eco-efficiency.

Years	Small Cities	Medium-Sized Cities	Large Cities	Mega-Cities	Super-Large Cities	Nationwide
2004	0.543	0.549	0.648	0.776	0.952	0.589
2005	0.587	0.606	0.701	0.833	0.970	0.643
2006	0.598	0.616	0.719	0.873	0.967	0.659
2007	0.607	0.616	0.731	0.862	0.965	0.667
2008	0.604	0.624	0.743	0.843	0.971	0.674
2009	0.634	0.639	0.751	0.877	0.966	0.692
2010	0.635	0.649	0.758	0.846	0.964	0.696
2011	0.636	0.676	0.767	0.846	1.002	0.714
2012	0.635	0.664	0.751	0.878	0.985	0.704
The overall average	0.609	0.627	0.730	0.848	0.971	0.671

**Table 3:** Estimated threshold effects and threshold values.

Threshold Variable	Thresholds	F-Statistic	*p*-Value	Critical Value			Threshold Value	95% Confidence Interval
				10%	5%	1%		
Spe	Single	81.020 ***	0.000	21.107	24.915	33.132	1.493	(1.482 1.495)
	Double	35.050 ***	0.000	21.343	23.196	28.709	1.632	(1.619 1.641)
	Triple	15.500	0.230	21.712	32.484	50.270	0.819	(0.814 0.822)
Rd	Single	35.880 ***	0.000	22.702	26.439	31.511	0.179	(0.166 0.186)
	Double	32.480 **	0.020	21.703	27.425	33.909	0.376	(0.367 0.378)
	Triple	15.870	0.670	36.014	40.670	48.171	0.856	(0.844 0.858)
Ud	Single	22.583 **	0.047	19.590	22.260	29.064	2.512	(2.347 2.517)
	Double	9.570	0.463	15.566	17.932	21.968	1.236	(1.209 1.266)
	Triple	9.860	0.343	14.763	17.235	22.416	2.257	(2.098 2.262)

Note: ** *p* < 0.05, *** *p* < 0.01.

**Table 4:** Results of the panel threshold model.

Variables	Coefficient	Variables	Coefficient	Variables	Coefficient
Spe[Spe ≤ 1.493]	−0.086 ***	Rd[Rd ≤ 0.179]	−0.026 **	Ud[Ud ≤ 2.512]	−0.082 ***
	(0.024)		(−0.013)		(0.028)
Spe[1.493 < Spe ≤ 1.632]	−0.028 **	Rd[0.179 < Rd ≤ 0.376]	0.029 **	Ud[Ud > 2.512]	−0.055 **
	(0.011)		(0.012)		(0.026)
Spe[Spe > 1.632]	0.029 **	Rd[Rd > 0.376]	0.098 ***		
	(0.012)		(0.024)		
Rd	0.039 **	Spe	−0.116 ***	Spe	−0.153 ***
	(0.016)		(0.034)		(0.034)
Ud	0.014	Ud	−0.032	Rd	−0.046 ***
	(0.027)		(0.026)		(0.016)
Er	−0.017 ***	Er	−0.017 ***	Er	−0.018 ***
	(0.006)		(0.006)		(0.006)
Is	−0.069 ***	Is	−0.065 ***	Is	−0.072 ***
	(0.023)		(0.023)		(0.023)
LnTech	0.050 ***	LnTech	0.051 ***	LnTech	0.046 ***
	(0.006)		(0.006)		(0.006)
Fdi	−0.008 ***	Fdi	−0.007 **	Fdi	−0.008 ***
	(0.003)		(0.003)		(0.003)
LnEdl	0.260 ***	LnEdl	0.246 ***	LnEdl	0.254 ***
	(0.012)		(0.012)		(0.012)
Gi	0.020	Gi	0.012	Gi	0.020
	(0.014)		(0.014)		(0.014)
C	−2.746 ***	C	−2.529 ***	C	−2.640 ***
	(0.123)		(0.123)		(0.124)
R^2^	0.5786	R^2^	0.6237	R^2^	0.5677
Obs	2547	Obs	2547	Obs	2547

Note: ** *p* < 0.05, *** *p* < 0.01; standard errors and threshold intervals are in parentheses and brackets, respectively.

**Table 5:** The selection test of spatial panel models.

Testing Method	Static SPDM			Dynamic SPDM		
	S_Spe	S_Rd	S_Ud	D_Spe	D_Rd	D_Ud
LM-lag test	44.693 ***	46.343 ***	43.096 ***	51.9995 ***	150.3003 ***	7.7305 ***
R-LM-lag test	35.947 ***	37.047 ***	34.722 ***	848.2076 ***	1299.1393 ***	27.0299 ***
LM-err test	80.738 ***	90.429 ***	76.978 ***	57,200 ***	19,500 ***	938.188 ***
R-LM-err test	71.992 ***	81.133 ***	68.604 ***	58,000 ***	20,700 ***	957.4873 ***
Hausman test	137.57 ***	101.04 ***	135.16 ***	26414.37 ***	35,169.33 ***	46,483.73 ***
LR-lag test	180.76 ***	39.39 ***	148.97 ***	19.08 **	18.75 **	18.61 **
LR-err test	178.45 ***	40.7 ***	146.48 ***	3496.82 ***	3444.38 ***	3505.87 ***
Wald-lag test	148.53 ***	18.2 **	84.64 ***	11,673.88 ***	8383.46 ***	4936.28 ***
Wald-err test	170.63 ***	18.16 **	116.18 ***	11,433.66 ***	8300.02 ***	4979.49 ***

Note: ** *p* < 0.05, *** *p* < 0.01.

**Table 6:** SPDM model results.

Variables	Static SPDM			Dynamic SPDM		
	S_Spe	S_Rd	S_Ud	D_Spe	D_Rd	D_Ud
Spe	−0.150 ***	−0.0704	0.0105	−2.557 ***	1.462 ***	1.153 ***
	(0.057)	(0.050)	(0.054)	(0.031)	(0.025)	(0.027)
Spe^2^	0.503 ***			3.893 ***		
	(0.102)			(0.054)		
Rd	0.250 ***	−0.244 ***	0.214 ***	−0.0347 ***	−0.351 ***	−0.141 ***
	(0.020)	(0.051)	(0.020)	(0.012)	(0.023)	(0.012)
Rd^2^		0.113 ***			0.419 ***	
		(0.020)			(0.012)	
Ud	−0.0932 *	−0.0963 **	−0.360 ***	2.325 ***	1.324 ***	0.698 ***
	(0.051)	(0.040)	(0.069)	(0.028)	(0.023)	(0.036)
Ud^2^			−0.120 **			−0.335 ***
			(0.059)			(0.031)
Er	−0.112 ***	−0.0121	−0.120 ***	0.127 ***	0.115 ***	0.056 ***
	(0.010)	(0.009)	(0.010)	(0.005)	(0.005)	(0.005)
Is	−0.0224	−0.212 ***	−0.00612	0.167 ***	0.268 ***	0.193 ***
	(0.022)	(0.037)	(0.022)	(0.011)	(0.011)	(0.011)
lnTech	−0.0279 ***	−0.00845	−0.0162 **	0.0352 ***	0.106 ***	0.0804 ***
	(0.008)	(0.009)	(0.008)	(0.004)	(0.004)	(0.004)
Fdi	0.018 ***	−0.009 **	0.014 ***	0.0210 ***	0.00858 ***	0.00665 ***
	(0.005)	(0.004)	(0.005)	(0.003)	(0.003)	(0.003)
lnEdl	0.087 ***	0.348 ***	0.102 ***	0.0658 ***	0.0385 ***	0.113 ***
	(0.017)	(0.028)	(0.018)	(0.009)	(0.009)	(0.009)
Gi	−0.106 ***	0.065 ***	−0.051 **	0.145 ***	0.502 ***	0.403 ***
	(0.025)	(0.024)	(0.024)	(0.013)	(0.012)	(0.012)
Iee(-1)				1.717 ***	1.869 ***	1.471 ***
				(0.015)	(0.015)	(0.015)
ρ	−0.368 *	−0.575 **	−0.386 *	9.945 ***	20.05 ***	8.391 ***
	(0.207)	(0.289)	(0.203)	(0.397)	(0.398)	(0.398)
R^2^	0.4199	0.3837	0.4333	0.3802	0.3974	0.4102
Obs	2547	2547	2547	2264	2264	2264

Note: * *p* < 0.1, ** *p* < 0.05, *** *p* < 0.01; standard errors are in parentheses. This table disregards the spatial lag coefficient of each explanatory variable due to the limited space.

**Table 7:** Threshold effect and threshold estimation results of city size.

Threshold Variable	Thresholds	F-Statistic	*p*-Value	Critical Value			Threshold Value	95% Confidence Interval
				10%	5%	1%		
Spe	Single	61.349 ***	0.000	32.252	38.342	44.610	79.910	(79.230 80.190)
	Double	17.000	0.450	27.326	33.364	47.217	34.540	(34.205 34.860)
	Triple	12.840	0.683	27.709	32.153	36.598	126.380	(125.005 128.020)
Rd	Single	69.237 ***	0.000	31.985	37.509	50.508	382.240	(375.435 392.874)
	Double	9.110	0.887	26.354	29.998	40.945	21.410	(20.525 22.230)
	Triple	7.520	0.877	22.513	24.985	33.873	31.910	(31.290 32.440)
Ud	Single	32.384 **	0.048	24.990	31.403	56.429	149.100	(144.320 149.710)
	Double	58.834 ***	0.002	30.008	37.336	47.113	497.150	(470.345 509.020)
	Triple	9.220	0.810	25.663	28.600	33.532	79.370	(77.915 79.660)

Note: ** *p* < 0.05, *** *p* < 0.01.

**Table 8:** Results of the panel threshold model (bootstrap = 300).

Variables	Coefficient	Variables	Coefficient	Variables	Coefficient
Spe[Us ≤ 79.910]	−0.014	Rd[Us ≤ 382.240]	0.078 ***	Ud[Us ≤ 149.100]	−0.023 **
	(0.024)		(0.027)		(0.010)
Spe[Us > 79.910]	−0.061 **	Rd[Us > 382.240]	0.134 ***	Ud[149.100 < Us ≤ 497.150]	−0.011 *
	(0.024)		(0.034)		(0.006)
				Ud[Us > 497.150]	0.015 *
					(0.008)
Rd	0.045 ***	Spe	−0.143 ***	Spe	−0.163 ***
	(0.016)		(0.034)		(0.034)
Ud	−0.042	Ud	−0.041	Rd	−0.049 ***
	(0.027)		(0.026)		(0.016)
Er	−0.018 ***	Er	−0.017 ***	Er	−0.019 ***
	(0.006)		(0.006)		(0.006)
Is	−0.074 ***	Is	−0.069 ***	Is	−0.074 ***
	(0.023)		(0.023)		(0.023)
LnTech	0.048 ***	LnTech	0.049 ***	LnTech	0.049 ***
	(0.006)		(0.006)		(0.006)
Fdi	−0.008 ***	Fdi	−0.009 ***	Fdi	−0.008 **
	(0.003)		(0.003)		(0.003)
LnEdl	0.255 ***	LnEdl	0.257 ***	LnEdl	0.260 ***
	(0.012)		(0.012)		(0.013)
Gi	0.020	Gi	0.018	Gi	0.019
	(0.014)		(0.014)		(0.014)
C	−2.671 ***	C	−2.678 ***	C	−2.678 ***
	(0.124)		(0.123)		(0.124)
R^2^	0.5472	R^2^	0.5259	R^2^	0.5359
Obs	2547		2547		2547

Note: * *p* < 0.1, ** *p* < 0.05, *** *p* < 0.01; standard errors are in parentheses; threshold intervals are in brackets.

## Data Availability

Not applicable.
